# Preoperative Low Lumbar Hounsfield Units and Global Alignment Predict Postoperative Mechanical Complications After Adult Spinal Deformity Surgery: A Multicenter Retrospective Study

**DOI:** 10.3390/jcm14124267

**Published:** 2025-06-16

**Authors:** Ippei Yamauchi, Hiroaki Nakashima, Sadayuki Ito, Naoki Segi, Jun Ouchida, Yoshinori Morita, Yukihito Ode, Yasuhiro Nagatani, Yuya Okada, Yosuke Takeichi, Yujiro Kagami, Hiroto Tachi, Kazuma Ohshima, Hiroki Oyama, Keisuke Ogura, Yuichi Miyairi, Ryotaro Oishi, Kazuaki Morishita, Ryuichi Shinjo, Tetsuya Ohara, Taichi Tsuji, Tokumi Kanemura, Shiro Imagama

**Affiliations:** 1 Department of Orthopedic Surgery, Nagoya University Graduate School of Medicine, Nagoya 466-8560, Japan; 2 Department of Orthopedics and Spine Surgery, Meijo Hospital, Nagoya 460-0001, Japan; 3 Department of Orthopedic Surgery, Toyota Kosei Hospital, Toyota 470-0396, Japan; 4 Department of Orthopedic Surgery, Konan Kosei Hospital, Konan 483-8704, Japan; 5 Department of Orthopedic Surgery, Anjo Kosei Hospital, Anjo 446-8602, Japan

**Keywords:** adult spinal deformity, Hounsfield unit, mechanical complications, multicenter study

## Abstract

**Objectives:** This study investigated the potential of Hounsfield unit (HU) values obtained from computed tomography (CT) scans as predictors of mechanical complications (MCs) in patients undergoing long-segment spinal fusion involving the pelvis. Additionally, it identified a threshold HU value associated with an increased risk of MCs. **Methods:** We conducted a retrospective, multicenter review of patients who underwent long-segment spinal fusion involving the pelvis, with a minimum follow-up period of two years. Patients were categorized based on the presence or absence of postoperative MCs. Both preoperative and postoperative radiographic parameters were analyzed, and HU values were quantified from CT images. Logistic regression modeling was used to identify independent risk factors for MCs. **Results:** Among 129 patients, 33 (25.6%) developed MCs, including proximal and distal junctional failures, rod fractures, and cases necessitating re-operation. The HU values were significantly lower in the MC group, whereas conventional bone mineral density (BMD) measurements showed no significant difference. Global alignment parameters, such as the sagittal vertical axis (SVA) and global tilt (GT), were consistently higher in patients with MCs. Receiver operating characteristic analysis identified 131 HU as the optimal cut-off, yielding a sensitivity of 56.4% and a specificity of 69.7%. Multivariate analysis confirmed that lower HU values were independently associated with the occurrence of MCs. **Conclusions:** Lower HU values and larger radiological global alignment parameters are significant predictors of MCs in patients undergoing surgery for adult spinal deformity. These findings underscore the importance of CT-based quantitative assessments in preoperative planning.

## 1. Introduction

The incidence of adult spinal deformity (ASD) increases with age, leading to pain and functional impairment and often necessitating corrective surgery [1,2,3]. Postoperative complications in ASD, including neurological disorders, hemorrhagic shock, hematoma, heart failure, and surgical site infections, are significantly higher in patients aged >65 years [4]. Osteoporosis, which is relatively common among older adults, is significantly associated with surgical outcomes in patients with ASD.

Recently, the focus has shifted to individual factors such as body mass index (BMI) and bone mineral density (BMD), along with alignment correction, to minimize postoperative complications [5,6,7]. BMD is typically assessed using dual-energy X-ray absorptiometry (DEXA), a widely adopted clinical tool. Nonetheless, its accuracy may be compromised in cases of spinal degeneration due to interference from osteophytes or surrounding soft tissues, which can lead to potential overestimation of bone density [8,9]. Consequently, studies have explored the usefulness of Hounsfield unit (HU) values measured by computed tomography (CT) as indicators of bone density [10,11,12,13]. HU values reflect the radiodensity of tissues based on their attenuation of X-rays and are influenced by both the density and the atomic composition at standard temperature and pressure for any planar region of interest (ROI). The attenuation of X-rays is proportional to the atomic mass and density of the irradiated tissue. Therefore, in the context of bone, HU values are proportional to mineral density [10]. Postoperative mechanical complications (MCs) such as proximal junctional kyphosis (PJK), proximal junctional failure (PJF), and screw loosening are more frequent in patients with lower HU values [11,14].

To the best of our knowledge, no multicenter study has yet examined the association between MCs and HU values or determined their cut-off values. Therefore, in this multicenter study, we aimed to investigate MCs following surgery for ASD and determine the cut-off HU values.

## 2. Materials and Methods

### 2.1. Patients

**Figure 1:**
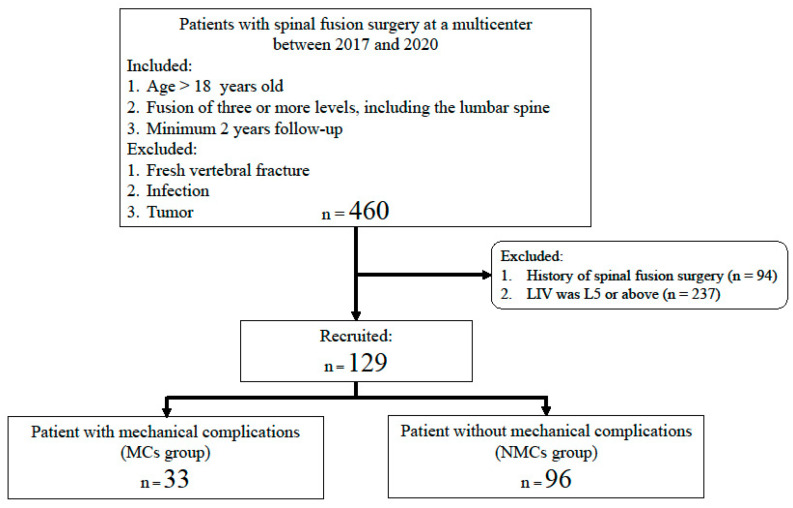
Flowchart of the study. LIV: lowest instrumented vertebra; MCs: mechanical complications; NMCs: no mechanical complications.

This multicenter, retrospective study included patients who underwent surgery for ASD from January 2017 to December 2020. Participants were eligible for inclusion if they were aged ≥18 years; had undergone fusion of at least three spinal segments, including the lumbar area; and had been monitored for a minimum of two years. Individuals were excluded from the study if they had a recent vertebral fracture, were suffering from an infection, or had a tumor present. Additionally, patients with a history of prior spinal operations and those with the lowest instrumented vertebra at L5 or above were also excluded. The study cohort was subsequently divided into two groups based on the presence or absence of postoperative MCs: a MC group and a non-complication group (NMC group) (Figure 1). For all included patients, relevant demographic and clinical information was obtained, including age, sex, body measurements (height, weight, and BMI), prior osteoporosis treatment history, operative details, and preoperative scores on the Japanese Orthopedic Association (JOA) scale for low back pain (0–29 points).

### 2.2. Radiographic Measurements

Standing anteroposterior and lateral whole-spine radiographs were evaluated at three different stages: before the surgery, immediately after the surgery, and two years following the surgery. The obtained measurements included the following:

- Thoracic kyphosis (TK: T1–12);
- Lumbar lordosis (LL: L1–S1);
- Lower lumbar lordosis (LLL: L4–S1);
- Pelvic incidence;
- Pelvic tilt;
- Sacral slope;
- C7 sagittal vertical axis (SVA);
- Global tilt (GT).

Global tilt was specifically defined as the angle formed by a line extending from the center of the C7 vertebra to the center of the sacral endplate, intersecting with a line drawn from the center of the femoral head to the center of the sacral endplate.

### 2.3. MCs

MCs included implant-related complications and adjacent segment disease, including PJK, PJF, and distal junctional failure (DJF). PJK was defined according to the criteria established by Glattes et al. [15]. PJF was defined as a fracture of the UIV or the vertebra immediately above the UIV, pullout of the screw at the UIV, and sagittal subluxation. DJF was defined as the loosening or pullout of the screw at the LIV.

### 2.4. HU Values Measurement

HUs were determined using an elliptical tool in standard picture archiving and communication system software. The ROI was determined as follows: the vertebral body with the least degeneration in the lumbar spine was selected, avoiding cortical bone, bone defects, and focal lesions, while selecting the largest possible oval site containing only trabecular bone.

### 2.5. Statistical Analysis

All statistical analyses were performed using IBM SPSS Statistics for Windows, version 22.0 (IBM Corp., Armonk, NY, USA). Continuous variables were summarized as means with standard deviations. Comparisons between two groups for continuous variables were conducted using the Mann–Whitney *U* test, while categorical variables were analyzed using Pearson’s chi-square test or Fisher’s exact test, as appropriate. To evaluate the discriminative ability of HU values in predicting MCs, receiver operating characteristic (ROC) curves were constructed, and the area under the curve (AUC) with 95% confidence intervals (CIs) was calculated. The optimal threshold value for HU was identified using Youden’s index, which reflects the best balance between sensitivity and specificity. Furthermore, a multivariate binary logistic regression model was employed to assess the independent predictors of MCs, incorporating potential confounders and variables that showed significant differences in univariate comparisons. A *p*-value of less than 0.05 was considered statistically significant.

## 3. Results

### 3.1. Baseline Patient Characteristics and Clinical Outcomes

This study included 129 patients: 33 in the MC group (84.8% female, 69.6 ± 7.8 years) and 96 in the NMC group (81.3% female, 67.4 ± 11.7 years). The MC group included 22, 2, 5, and 9 cases of PJK/PJF, DJF, rod fracture, and requiring re-operation, respectively. Reoperations included five PJK/PJF, one DJF, and three rod fractures. Baseline characteristics, including age, sex, anthropometric data (height, weight, and BMI), and surgical details, were comparable between the MC and NMC groups, with no statistically significant differences (Table 1). Twenty-three patients were treated for osteoporosis preoperatively, with most of them in the MC group. Japanese Orthopedic Association scores for low back pain were similar across both groups. However, at the 2-year follow-up, the NMC group demonstrated significantly better scores compared with the MC group (MC group, 20.7 ± 3.9; NMC group, 23.3 ± 3.7; *p* < 0.01).

### 3.2. Comparison Radiographic Measurements Between MC and NMC Groups

Table 2 presents the results of the radiographic evaluations. The MC group exhibited a significantly larger SVA prior to surgery compared with the NMC group (101.3 ± 49.5 vs. 79.8 ± 56.9; *p* = 0.03). Differences in GT were also apparent at multiple time points following surgery, immediately after surgery (MC: 43.7 ± 37.2, NMCs: 29.1 ± 31.8; *p* < 0.04) and at the 2-year follow-up (MC: 71.2 ± 53.9, NMCs: 45.2 ± 45.8; *p* = 0.01). Additionally, the MC group demonstrated significantly greater GT values before surgery (42.5 ± 23.1 vs. 31.5 ± 18.9; *p* = 0.03), and this trend persisted at 2 years postoperatively (33.3 ± 18.4 vs. 22.2 ± 16.7; *p* < 0.01). No other spinopelvic alignment parameters showed statistically significant differences between the two groups at any evaluated time point.

### 3.3. BMD Measurements Using HUs

**Figure 2:**
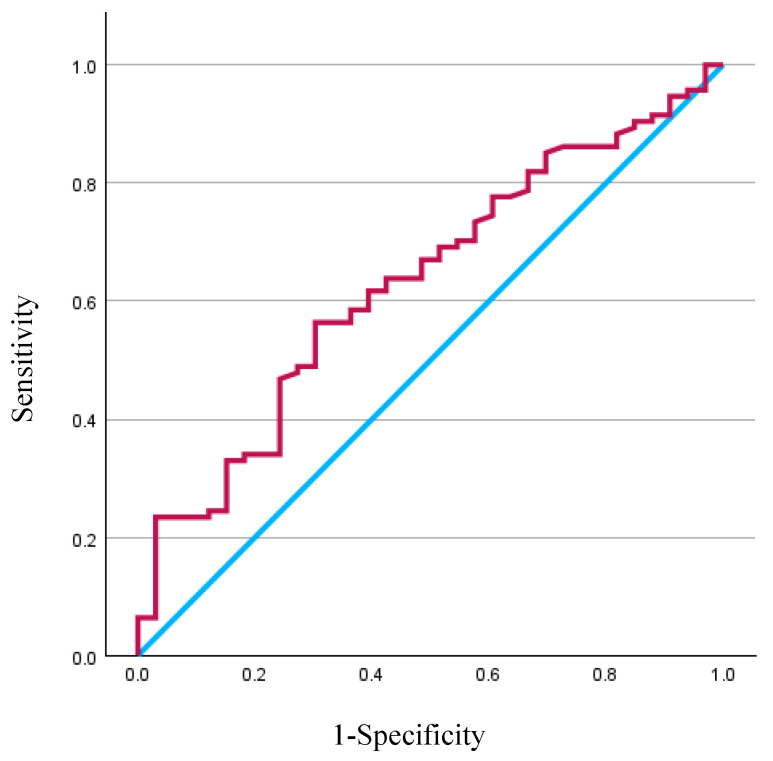
Receiver operating characteristic (ROC) curves for lumbar Hounsfield unit (HU) values in predicting mechanical complications (MCs) showed an area under the curve (AUC) of 0.631 (95% confidence interval, 0.525–0.737). The optimal cut-off for HU values to distinguish between patients with MCs and those without was 131, achieving a sensitivity of 56.4% and a specificity of 69.7%.

HUs were significantly lower in the MC group (116.3 ± 39.4) compared with the NMC group (138.5 ± 49.5; *p* = 0.03). ROC analysis revealed that HU values had a significant predictive capacity for MCs, with an AUC of 0.631 (95% CI, 0.525–0.737; *p* = 0.016) (Figure 2). The optimal HU cut-off value for predicting MCs was 131, with a sensitivity and specificity of 56.4% and 69.7%, respectively.

### 3.4. Multivariate Predictor of MCs

The results of the multivariate logistic regression analysis, which examined the independent predictors of MCs, including baseline parameters such as age and sex, along with the preoperative radiological parameter SVA, revealed significant differences found in the univariate analysis. HU values are presented in Table 3. Both GT and SVA are global alignment parameters and are subject to many confounding factors. Therefore, we opted for SVA, which is the most commonly used parameter. For every one-point increase in HU value, the probability of developing MCs increased 0.997-fold (*p* = 0.046).

### 3.5. Case Presentation

**Figure 3:**
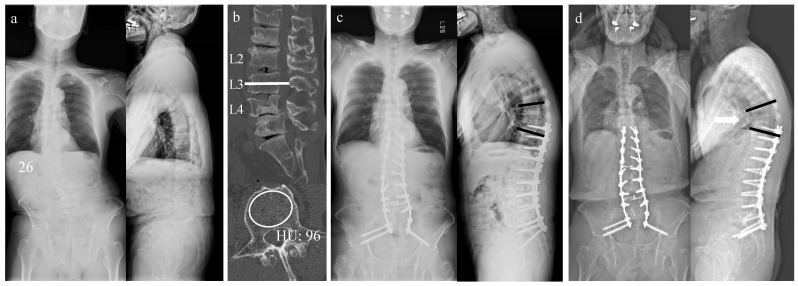
Representative case: (**a**) Preoperative standing whole-spine anteroposterior and lateral views. (**b**) Preoperative computed tomography views. (**c**) Immediate postoperative standing sagittal and lateral views. (**d**) Two years postoperative standing sagittal and lateral views. Black line means proximal junctional angle. The white arrows indicate the occurrence of PJK.HU: Hounsfield unit; PJK: proximal junctional kyphosis.

Figure 3 illustrates a representative case of the MC group. A 76-year-old male presented with lower back and bilateral leg pain. Whole-spine radiography revealed 26-degree lumbar scoliosis (Figure 3a). The HU value in the L3 vertebral axial view (Figure 3b) was 96. In contrast, DEXA measurements of BMD showed normal values in the right femoral neck (BMD, 0.817 g/cm^2^; T-score, −1.0; mean percentage of young adults, 87%) and lumbar spine (BMD, 1.435 g/cm^2^; T-score, 2.0; mean percentage of young adults, 121%). The patient underwent corrective anterior and posterior spinal fusion surgery from T10 to the pelvis (Figure 3c). Radiography revealed PJK 2 years postoperatively (Figure 3d, PJK: white arrow).

## 4. Discussion

This multicenter investigation is, to the best of our knowledge, the first to demonstrate that reduced preoperative HU values are associated with a higher incidence of MCs following ASD surgery, with 131 identified as the optimal threshold. BMD was not associated with MCs, suggesting that BMD assessment in a degenerated spine may not accurately reflect bone strength and may, therefore, be unrelated to MCs. Additionally, consistent with the findings of previous studies, other factors such as SVA and GT, which represent radiological global alignment, were significantly greater in patients who experienced MCs both preoperatively and after corrective surgery [16,17,18,19].

The incidence of ASD increases with age; however, it has also risen in parallel with increased life expectancy over recent years [1]. ASD results in pain, functional limitations, and reduced health-related quality of life [2,3]. As corrective surgery for ASD improves health-related quality of life, the number of patients undergoing such procedures continues to rise [20]. However, surgical treatment for ASD remains challenging due to the invasiveness of the procedure and the fact that many patients, particularly older individuals, have multiple comorbidities and poor general health. In a systematic review, Zanirato et al. reported that complications occurred in 37% of patients, with neurological disorders, infectious complications, and MCs being the most common [21]. Minimizing postoperative complications remains a critical concern in current corrective surgeries for ASD.

Correcting proper alignment is one of the most important surgical factors for reducing postoperative complications. In 2012, Schwab et al. reported the importance of alignment in the Scoliosis Research Society–Schwab ASD classification, which is now widely used [22]. A postoperative SVA of <50 mm is considered an indicator of successful alignment as restoration of the SVA facilitates horizontal gaze and supports a physiologically comfortable standing posture [23]. Banno et al. reported that GT, as a global alignment parameter, increases with age and is correlated with health-related quality of life [16]. The Global Alignment and Proportion (GAP) score was developed to reduce the incidence of postoperative MCs by grading pelvic incidence (PI), age, and four radiological factors [24]. However, the predictive accuracy of the GAP score for MCs remains a topic of debate, likely due to the influence of not only spinal alignment but also patient-specific factors such as BMI and BMD [5,6,17,19]. To address this limitation, Noh et al. introduced the GAPB score, which extends the original model by incorporating both BMI and BMD. Among these variables, BMD has gained particular attention as a key factor contributing to the risk of postoperative complications [18]. Williamson et al. also identified obesity and bone density as parameters that can be modified preoperatively [7].

DEXA remains the most commonly used method for evaluating BMD; however, its reliability can be compromised under certain clinical conditions, particularly in the presence of spinal degeneration or structural changes. Masud et al. reported that even mild osteophytes can result in falsely elevated BMD measurements in the lumbar spine [8], while Svendsen et al. found that soft tissues may interfere with accuracy [9]. Several recent studies have explored the use of CT-based HU values as an alternative for assessing bone density.

Among individuals with degenerative changes in the spine, BMD assessments using DEXA may not accurately reflect true bone strength, as structural alterations, such as osteophytes or calcified ligaments, can lead to artificially elevated T-scores. By contrast, CT-derived HU values have been increasingly recognized as a more reliable indicator of vertebral bone quality in such populations. Lee et al. also reported a positive correlation between HU values and bone strength in finite element analysis [25].

Previous research has suggested that HU values below approximately 110–135 are indicative of osteoporotic bone, offering a more nuanced assessment than standard densitometry techniques [10,12,13]. Age-related decline in vertebral HU values has also been well characterized; for instance, normative data indicate that HU values in healthy young adults may exceed 200, while values in older adults, especially those over 80, often drop below 80, with a notable decline beginning after the sixth decade of life [26].

Several clinical studies have investigated the relationship between preoperative HU values and MCs following corrective spinal procedures. These studies consistently reported that lower HU values were associated with a higher incidence of complications such as PJK or pedicle screw loosening. One study reported significantly lower HU values in patients who developed PJK, independent of global alignment scores [11], while another identified a threshold of 108 HU for predicting screw instability with moderate accuracy [14].

Various methods of measuring HU values have been reported [11,13,14,26]. Hiyama et al. measured HU values at the UIV and at UIV + 1 and UIV + 2 vertebrae; they reported the association between the occurrence of PJF and HU values in ASD. The mean HU values at the UIV were significantly lower in the PJF group (PJF; 116.6 ± 28.1) than in the non-PJF group (141.8 ± 41.8, *p* = 0.049) [11]. Choi et al. reported a correlation between HU values measured at the midline of the L1–4 vertebrae and BMD [13]. Ishikawa et al. reported an association between sacral HU values and S1 screw loosening in patients with lumbosacral fusion, with the advantage of being able to measure at any location (loosening group, 62 ± 43; non-loosening group, 123 ± 63) [27]. Uei et al. reported that HU values are different for each vertebra; therefore, measuring vertebrae where complications may occur seems logical. However, complications such as rod fracture loss and pseudoarthrosis may be related to overall bone loss, and further investigation is needed [28]. Given the variability in HU measurement techniques, we adopted a streamlined and reproducible method: selecting a single lumbar vertebra with minimal degeneration and measuring HU within its trabecular region while avoiding cortical bone margins. This approach proved effective, yielding consistent values with minimal inter-case variability.

In the present study, we identified 131 HU as the cut-off value most closely associated with the occurrence of postoperative MCs. This value was consistent with previous reports and reinforced the clinical relevance of HU-based assessments. Notably, unlike DEXA, HU measurement was less likely to be confounded by age-related or disease-induced skeletal changes, making it a valuable adjunct in preoperative evaluation despite the need for CT imaging. Additionally, our results underscored the importance of spinopelvic alignment. Patients with larger preoperative and postoperative SVA and GT values were more likely to experience complications. The combination of poor sagittal balance and low vertebral HU values may serve as a strong predictor of surgical risk, suggesting that enhanced preoperative optimization, in terms of both alignment correction strategies and bone health management, may be warranted in high-risk individuals. However, the AUC determined using univariate analysis was 0.63, which is not a high predictive performance. This suggests that it is difficult to completely predict MCs by HU value alone. MCs in ASD are known to be associated with multiple factors, including age, BMI, paraspinal muscles, and alignment, and the identification of multifactorial complication factors, including HU values, is desirable [18].

The primary limitation of this study was its retrospective design. Prospective patient selection and standardization of treatment protocols would improve the reliability of the findings. Moreover, a comparative study controlling for factors such as smoking, diabetes, and corticosteroid use would have been desirable; however, this was not possible due to the small sample size. Additionally, the minimum follow-up period was only two years; therefore, studies with longer follow-up durations are desirable. Furthermore, the impact of complications on patient outcomes was not evaluated and should be addressed in future research. Nevertheless, this multicenter study demonstrated an association between HU values and postoperative complications following ASD surgery and identified a clinically relevant HU cut-off.

## 5. Conclusions

In conclusion, MCs occurred in 25.6% of patients following ASD surgery. Patients in the MC group had significantly greater global alignment parameters (SVA and GT) both preoperatively and postoperatively, as well as significantly lower HU values. Surgical planning that includes appropriate alignment correction and osteoporosis treatment may be necessary for patients with poor preoperative global alignment and low HU values.

## Figures and Tables

**Table 1:** Patient baseline characteristics and clinical outcomes. Data are presented as the mean ± standard deviation.

	Total	MC Group		NMC Group	*p*-Value
Age (years)	68.0 ± 10.9	69.6 ± 7.8		67.4 ± 11.7	NS
Sex (female, %)	82.2	84.8		81.3	NS
Height (cm)	152.4 ± 8.2	150.5 ± 7.0		153.1 ± 8.4	NS
Weight (kg)	55.8 ± 11.7	54.1 ± 11.2		56.4 ± 11.9	NS
BMI (kg/cm^2^)	23.9 ± 4.0	23.8 ± 4.2		23.9 ± 4.0	NS
Osteoporosis treatment PTH Bisphosphonate RANKL inhibitor	12 10 1	6 6 0		6 4 1	0.02
Operative parameters					
Time (minutes)	268.9 ± 111.2	257.2 ± 106.0		273.0 ± 113.2	NS
Blood loss (g)	1057.1 ± 1388.4	896.4 ± 1127.1		1112.4 ± 1468.8	NS
Fusion level (n)	6.5 ± 2.9	6.5 ± 3.3		6.5 ± 2.8	NS
UIV T2-5 T10-L3	10 119	3 30		7 89	NS
LIV Sacrum Ilium	18 111	6 27		12 84	NS
Duration of hospital stay (day)	52.8 ± 37.3	46.4 ± 32.2		55.1 ± 38.9	NS
JOA score					
Pre	15.4 ± 5.0	14.6 ± 4.8		15.7 ± 5.1	NS
2 years postoperatively	22.6 ± 3.9	20.7 ± 3.9		23.3 ± 3.7	<0.01
MCs		PJK/PJF	22		
		DJF	2		
		Rod fracture	5		
		Re-operation	9		

BMI: body mass index; PTH: para thyroid hormone; RANKL: receptor activator of nuclear factor-kappa B ligan; UIV: upper instrumented vertebra; LIV: lowest instrumented vertebra; DJF: distal junctional failure; JOA: Japanese Orthopedic Association; MCs: mechanical complications; MD: moderately disproportioned; NMCs: no mechanical complications; NS: not significant; PJF: proximal junctional failure; PJK: proximal junctional kyphosis; SD: severely disproportioned.

**Table 2:** Patient radiographic parameters.

Degrees	Total	MC Group	NMC Group	*p*-Value
TK				
Pre	23.6 ± 15.9	25.1 ± 16.9	23.0 ± 15.6	NS
Post-op	30.2 ± 12.0	29.4 ± 13.6	30.5 ± 11.4	NS
2 years post-op	35.6 ± 13.0	37.5 ± 17.8	34.9 ± 11.1	NS
LL				
Pre	23.6 ± 31.3	20.4 ± 18.7	24.7 ± 34.6	NS
Post-op	42.4 ± 11.8	42.0 ± 12.4	42.5 ± 11.6	NS
2 years post-op	38.8 ± 13.5	35.7 ± 14.2	40.0 ± 13.2	NS
LLL				
Pre	18.1 ± 13.8	15.0 ± 12.1	19.1 ± 14.3	NS
Post-op	26.4 ± 7.6	24.8 ± 9.7	27.0 ± 6.8	NS
2 years post-op	27.2 ± 18.9	24.7 ± 7.7	28.1 ± 21.5	NS
PI				
Pre	52.4 ± 12.3	53.2 ± 10.7	52.1 ± 12.9	NS
Post-op	49.9 ± 12.1	49.1 ± 14.1	50.2 ± 11.4	NS
2 years post-op	52.6 ± 13.4	53.1 ± 13.6	52.4 ± 13.4	NS
PT				
Pre	29.9 ± 12.1	33.0 ± 11.8	28.9 ± 12.1	NS
Post-op	20.5 ± 8.5	20.4 ± 8.3	20.5 ± 8.6	NS
2 years post-op	25.0 ± 9.5	27.3 ± 9.5	24.2 ± 9.4	NS
SS				
Pre	22.5 ± 13.3	20.2 ± 14.1	23.2 ± 13.0	NS
Post-op	29.4 ± 10.2	28.7 ± 11.5	29.7 ± 9.8	NS
2 years post-op	27.6 ± 13.5	25.7 ± 14.9	28.2 ± 12.9	NS
PI-LL				
Pre	30.9 ± 19.9	32.8 ± 17.5	30.3 ± 20.7	NS
Post-op	7.5 ± 77.7	7.1 ± 13.4	7.6 ± 11.2	NS
2 years post-op	14.0 ± 14.7	17.3 ± 17.3	12.8 ± 13.6	NS
SVA				
Pre	85.5 ± 55.7	101.3 ± 49.5	79.8 ± 56.9	0.03
Post-op	32.9 ± 33.7	43.7 ± 37.2	29.1 ± 31.8	0.04
2 years post-op	51.7 ± 49.0	71.2 ± 53.9	45.2 ± 45.8	0.01
GT				
Pre	34.4 ± 20.6	42.5 ± 23.1	31.5 ± 18.9	0.03
Post-op	20.3 ± 15.1	24.6 ± 18.2	18.9 ± 13.6	NS
2 years post-op	25.1 ± 17.8	33.3 ± 18.4	22.2 ± 16.7	<0.01

GT: global tilt; LL: lumbar lordosis; LLL: lower lumbar lordosis; MCs: mechanical complications; NMCs: no mechanical complications; NS: not significant; PI, pelvic incidence; PT, pelvic tilt; SS, sacral slope; SVA: sagittal vertical axis; TK, thoracic kyphosis.

**Table 3:** Multivariate predictor of MCs.

Predictors	Odds Ratio	Standard Error	*p*	95% CI
Age	0.997	0.027	0.926	0.946–1.052
Sex	0.933	0.593	0.906	0.291–2.985
SVA	1.007	0.004	0.097	0.999–1.014
HU value	0.989	0.005	0.046	0.979–1.000

CI: confidence interval; SVA: sagittal vertical axis; HU: Hounsfield unit.

## Data Availability

The original contributions presented in this study are included in the article. Further inquiries can be directed to the corresponding author.

## Abbreviations

The following abbreviations are used in this manuscript:

ASD	Adult spinal deformity
AUC	Area under the curve
BMD	Bone mineral density
BMI	Body mass index
CI	Confidence interval
CT	Computed tomography
DEXA	Dual-energy X-ray absorptiometry
DJF	Distal junctional failure
GAP	Global alignment and proportion
GT	Global tilt
HU	Hounsfield unit
MC	Mechanical complications
PJF	Proximal junctional failure
PJK	Proximal junctional kyphosis
ROI	Region of interest
SVA	Sagittal vertical axis
UIV	Upper instrumented vertebra
